# Enzyme-Loaded Flower-Shaped Nanomaterials: A Versatile Platform with Biosensing, Biocatalytic, and Environmental Promise

**DOI:** 10.3390/nano11061460

**Published:** 2021-05-31

**Authors:** Khadega A. Al-Maqdi, Muhammad Bilal, Ahmed Alzamly, Hafiz M. N. Iqbal, Iltaf Shah, Syed Salman Ashraf

**Affiliations:** 1 Department of Chemistry, College of Science, UAE University, Al Ain P. O. Box 15551, United Arab Emirates; 200935138@uaeu.ac.ae (K.A.A.-M.); ahmed.alzamly@uaeu.ac.ae (A.A.); 2 School of Life Science and Food Engineering, Huaiyin Institute of Technology, Huaian 223003, China; bilaluaf@hotmail.com; 3 Tecnologico de Monterrey, School of Engineering and Sciences, Monterrey 64849, Mexico; hafiz.iqbal@tec.mx; 4 Department of Chemistry, College of Arts and Sciences, Khalifa University, Abu Dhabi P. O. Box 127788, United Arab Emirates

**Keywords:** hybrid nanoflowers, biosynthesis, influencing factors, biosensing cues, bio-catalysis

## Abstract

As a result of their unique structural and multifunctional characteristics, organic–inorganic hybrid nanoflowers (hNFs), a newly developed class of flower-like, well-structured and well-oriented materials has gained significant attention. The structural attributes along with the surface-engineered functional entities of hNFs, e.g., their size, shape, surface orientation, structural integrity, stability under reactive environments, enzyme stabilizing capability, and organic–inorganic ratio, all significantly contribute to and determine their applications. Although hNFs are still in their infancy and in the early stage of robust development, the recent hike in biotechnology at large and nanotechnology in particular is making hNFs a versatile platform for constructing enzyme-loaded/immobilized structures for different applications. For instance, detection- and sensing-based applications, environmental- and sustainability-based applications, and biocatalytic and biotransformation applications are of supreme interest. Considering the above points, herein we reviewed current advances in multifunctional hNFs, with particular emphasis on (1) critical factors, (2) different metal/non-metal-based synthesizing processes (i.e., (i) copper-based hNFs, (ii) calcium-based hNFs, (iii) manganese-based hNFs, (iv) zinc-based hNFs, (v) cobalt-based hNFs, (vi) iron-based hNFs, (vii) multi-metal-based hNFs, and (viii) non-metal-based hNFs), and (3) their applications. Moreover, the interfacial mechanism involved in hNF development is also discussed considering the following three critical points: (1) the combination of metal ions and organic matter, (2) petal formation, and (3) the generation of hNFs. In summary, the literature given herein could be used to engineer hNFs for multipurpose applications in the biosensing, biocatalysis, and other environmental sectors.

## 1. Introduction

Enzymes are proteins (or ribonucleic acids) that act as catalysts to quicken chemical reactions by decreasing the activation energy. They are environmentally friendly and do not alter or get consumed during chemical reactions. Enzymes as biocatalysts have recently become an intensive area of research. Currently, enzymes are used in many industrial applications, including food, drugs, and water remediation [1,2,3,4,5,6,7], and have numerous benefits, including a high catalytic efficiency, a high selectivity, and biodegradability. Despite all of these benefits, the use of enzymes in industrial applications has some limitations, such as a low operational stability, difficult recovery, low reproducibility, and a high cost. Immobilization of the enzyme on an insoluble solid support has been found to be a useful way to overcome some of these limitations. The solid support must be inert, insoluble, nontoxic, environmentally safe, easily accessible, affordable, highly resistant to decay and microbial attack, and have an affinity to the enzyme used. Enzyme immobilization has many advantages, including functional stability, stability against extreme reaction conditions (changes in the pH and temperature), reusability, easy separation and recovery of enzymes, and increased catalytic performance. Many researchers have shown that immobilized enzymes are more stable than free enzymes. However, in some instances, immobilization can limit the enzyme performance and lower its catalytic activity. The reasons for this are the blocking of the active site on the enzyme and the conformational changes that happen to the enzyme after immobilization, as well as the limitations of mass transfer [8,9,10,11,12,13,14]. Therefore, there is a need to develop new and unique methods and materials to overcome these shortcomings caused by traditional immobilization methods. There is currently growing interest in the use of nanoscale materials, such as nanoparticles and nanocrystals, for enzyme immobilization [15,16,17,18,19].

More recently, Jun Ge et al. developed a new method of immobilizing enzymes on solid supports, known as organic–inorganic hybrid nanoflowers or hybrid nanoflowers, (hNFs), which are flower-like hybrid nanomaterials produced between a metal node and a protein through coordination interactions [20]. This review will focus on enzyme immobilization using organic–inorganic hybrid nanoflowers (hNFs), and will cover their synthesis, advantages, different types of nanoflowers, and applications.

## 2. Organic–Inorganic Hybrid Nanoflowers

**Figure 1:**
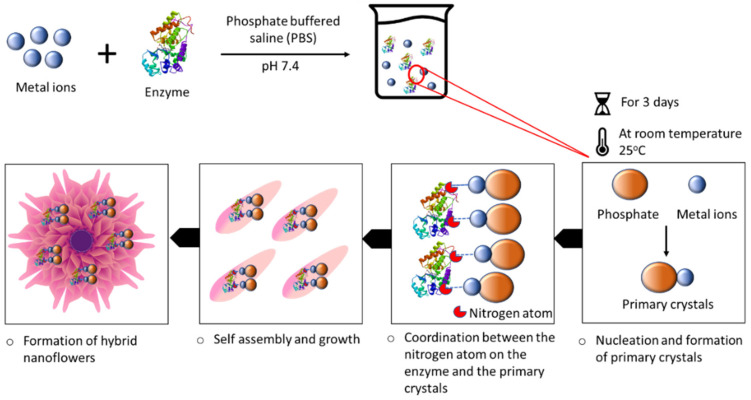
Schematic illustration of the hybrid nanoflower (hNF) formation process.

**Figure 2:**
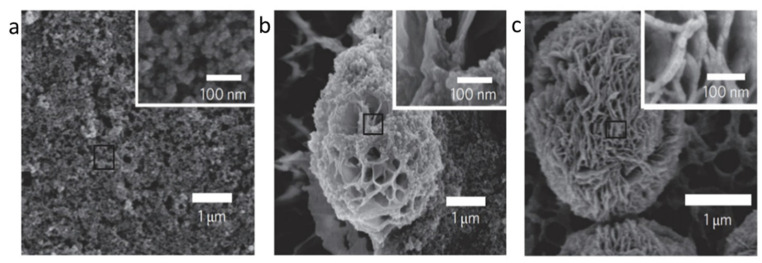
Formation of BSA-Cu_3_(PO_4_)_2_.3H_2_O nanoflowers. The SEM images at different times: (**a**) 2 h, (**b**) 12 h, and (**c**) 3 days. Reprinted from [20] with permission from Springer Nature. Copyright © 2021, Nature Publishing Group. License Number: 5031780508604.

Organic–inorganic hybrid nanoflowers were accidentally discovered in 2012. hNFs were first detected when 0.8 mM CuSO4 was added to phosphate-buffered saline (PBS) with 0.1 mgml^−1^ bovine serum albumin (BSA). The reaction pH was 7.4 at 25 °C. After 3 days, a blue precipitate was formed at the bottom of the reaction tube, resembling a flower structure. The formation of the organic–inorganic hNFs was confirmed using scanning electron microscopy (SEM) and transmission electron microscopy (TEM) [20]. The formation mechanism (self-assembly) of organic–inorganic hybrid nanoflowers occurs through the following three stages (Figure 1): nucleation, growth, and completion. In the first step, the nucleation of primary crystals is formed from protein molecules and Cu^2+^ ions. This occurs through binding to the amide group onto the protein backbone. The second step is the growth of seed particles of the Cu^2+^ binding site, which leads to the production of nano petals. This continues with protein nanoparticles and primary crystals. In the last step, the formation of organic–inorganic hybrid nanoflowers is completed. Figure 2 shows the SEM images of the organic–inorganic hybrid nanoflower formation after 2 h, 12 h, and 3 days [20].

**Figure 3:**
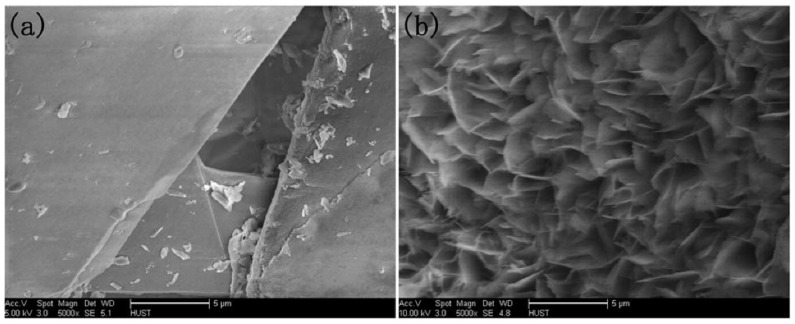
SEM image (**a**) without the addition of lipase enzyme, (**b**) with the addition of lipase enzyme. Reprinted from [21] with permission from the Royal Society of Chemistry. Copyright © The Royal Society of Chemistry. License Number: 1105113-1.

**Figure 4:**
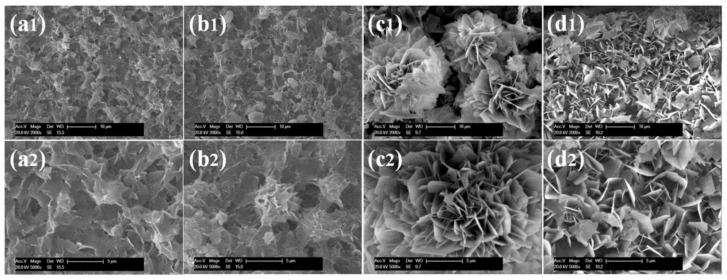
SEM images of different ChT concentrations on the formation of nanoflowers: (**a1**,**a2**) 0.0 mg/mL, (**b1**,**b2**) 0.05 mg/mL, (**c1**,**c2**) 0.1 mg/mL, and (**d1**,**d2**) 0.5 mg/mL. Reprinted from [22] with permission from the Royal Society of Chemistry. Copyright © The Royal Society of Chemistry. License Number: 1105121-1.

**Figure 5:**
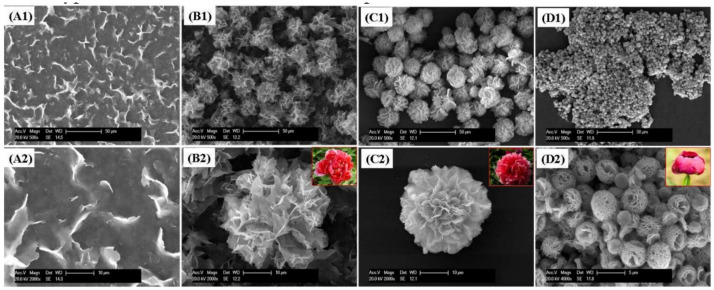
SEM image of different trypsin concentrations on the formation of nanoflowers: (**A1**,**A2**) 0.0 mg/mL, (**B1**,**B2**) 0.02 mg/mL, (**C1**,**C2**) 1.0 mg/mL, and (**D1**,**D2**) 5.0 mg/mL. Reprinted from [23] with permission from the Royal Society of Chemistry. Copyright © The Royal Society of Chemistry. License Number: 1105124-1.

**Figure 6:**
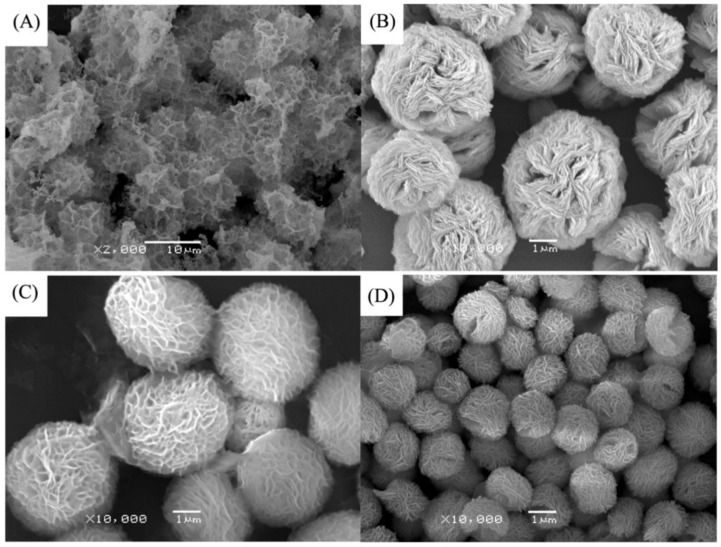
SEM image of different soybean peroxidase (SBP) concentrations on the formation of nanoflowers: (**A**) 0.0 mg/mL, (**B**) 0.5 mg/mL, (**C**) 1.0 mg/mL, and (**D**) 2.0 mg. Reprinted from [24] with permission from Elsevier. Copyright © 2021 Elsevier B.V. License Number: 5031790214453.

Additional research on organic–inorganic hybrid nanoflowers shows that the presence of a protein (enzyme) is essential for the formation of nanoflowers, and without an enzyme, only a crystal structure is formed. A research group formed hNFs between *Burkholderia cepacia* lipase (BCL) and calcium phosphate, and a part of their results (Figure 3) shows that the morphology transforms from sheet stacking to flower-like after the addition of lipase [21]. Another research group used α-chymotrypsin (ChT) and calcium phosphate to form hNFs, testing the effect of different enzyme concentrations on the formation of hNFs (Figure 4). It is clear from the SEM image that for samples a1 and a2, where ChT was not added, there is no formation of nanoflowers, and only the presence of large crystals are observed. However, when 0.05 mg/mL ChT was added, small buds were observed (b1 and b2), and as the enzyme concentration increased, nanoflower formation was more evident [22]. Lin et al. immobilized trypsin on hNFs and studied its application as a reactor for highly efficient protein digestion. One of the experiments they ran shows that for hNFs to be formed, the trypsin enzyme must be added, and without trypsin, large crystals are formed (Figure 5). The figure also shows that with an increasing trypsin concentration, a flower-like structure appears [23]. Researchers in another study produced hNFs from soybean peroxidase (SBP) and Cu^2+^. Their outcomes showed the same result as previous work, where the absence of a protein (SBP) produced a crystal-like structure, but not nanoflowers (Figure 6) [24]. Moreover, their research shows that the presence of proteins in the formation of hybrid nanoflowers is crucial.

Currently the majority of enzyme immobilization techniques use preexisting carriers. In such a case, the enzyme can be immobilized by physical adsorption or attachment through covalent bonds between the carrier and the enzyme. These methods usually consist of the following two stages: one is the synthesis of the carriers, and the second is the immobilization process of the enzyme on the carrier. These two steps can cause a reduction in the reaction efficiency. Moreover, they require a lengthier process, which results in a higher cost. One factor that distinguishes hNFs from other immobilization techniques is that it is a one-step reaction, i.e., the carrier synthesis and enzyme immobilization processes occur in one step. This leads to a simpler procedure, and thus a lower cost [25].

## 3. Advantages or (Properties) of Hybrid Nanoflowers

### 3.1. Catalytic Activity

Several enzymes, including carbonic anhydrase, lipase, trypsin, laccase, and different types of peroxidases, have been used for the formation of hNFs through immobilization techniques [23,24,26,27,28,29,30]. A significant advantage of hNF formation over other immobilization methods is the increase in the immobilized enzyme’s catalytic activity [24]. Studies have shown that immobilization only improves the stability, not the catalytic activity of the enzyme (with rare exceptions). This could be as a result of the mass transfer limitations and conformational changes in the enzyme [31,32]. For hNFs, the enhancement of the catalytic activity of the immobilized enzyme possibly arises from several reasons, namely: (i) the high surface area of hNFs, (ii) less mass transfer limitation, (iii) cooperative effect of the nanoscale-entrapped enzyme, and (iv) favorable enzyme conformation in hNFs [31,33,34].

A study showed that the enzymatic activities of soybean peroxidase in hNFs formed from different concentrations of crude SBP (0.5, 1, and 2 mg/mL) were 787, 1857, and 2500 U/mg, respectively. These results showed a ~137%, ~325%, and ~446% increase in activity compared with free crude SBP, respectively, which has an activity of 572 U/mg [24]. Another study produced hNFs and magnetic hNFs with laccase enzyme and copper(II) sulfate pentahydrate (CuSO_4_.5H_2_O). Laccase showed a higher activity in both hNFs than free laccase. The laccase hNF activity was 3.3 times greater than that of free laccase, and the laccase magnetic hNF activity was 2.7 times greater than that of free laccase. The reduction in activity in the magnetic hNFs was attributed to the shielding of active sites on laccase by the magnetic nanoparticles on the surface of the hNFs [34]. Another research group working with lipase enzymes found that lipase/Zn_3_(PO_4_)_2_ hNFs had a higher enzyme activity (855 ± 13 U/g) than free lipase (328 ± 6 U/g). The increase in the enzyme activity was 147% of the pure enzyme [35]. Yin et al. synthesized α-chymotrypsin (ChT) hNFs and studied their use as immobilized ChT reactors for successful protein digestion. They determined the ChT activity in hNFs to be 3410 U/mg compared with free ChT, which has an activity of 1123 U/mg. The results show that the enhancement in the ChT activity was approximately 266% higher [22]. Lin et al., in another study, synthesized hNFs using horseradish peroxidase enzyme (HRP) and copper phosphate (Cu_3_(PO_4_).3H_2_O) to use as a colorimetric platform for the visual identification of phenol and hydrogen peroxide. The results obtained showed a considerable improvement in the activity of the embedded HRP enzyme in the nanoflowers. The free HRP activity was 2970.5 U/mg, whereas the embedded HRP activity was 15,040.5 U/mg. This led to a 506% increase in the inactivity of the HRP-embedded nanoflowers [36]. All of these studies solidified that organic–inorganic hybrid nanoflowers significantly boost the catalytical activity of the embedded enzyme, which can be ascribed to the four previously-mentioned reasons.

### 3.2. Stability

#### 3.2.1. Thermal Stability

Another advantage of hNFs is the stability they provide to the enzyme. Regarding thermal stability, a research group, Yu et al., studied the temperature effect on hNFs formed using calcium phosphate and six different enzymes, namely: papain, bromelain, trypsin, lipase from porcine pancreas (PPL), lipase from *Thermomyces lanuginosus* (TLL), and lipase B from *Candida antarctica* (CALB). The results were tested in different temperature ranges of 50, 60, and 70 °C, and showed that all enzyme-hNFs were more thermally stable than their corresponding free enzymes. For example, after heating at 70 °C for 6 h, the residual activity of the enzymes in hNFs was as follows: TLL-hNFs (78.3%), PPL-hNFs (72.9%), and CALB-hNFs (84.3%), counting for a 4.57, 2.61, and 2.35 times higher activity than the corresponding free enzymes, respectively. The authors attributed this stability to the strong interaction between the Ca^2+^ ions and the functional groups on the enzymes in the hNFs and the rigidity in the inorganic hNFs that enclosed the enzyme and stopped the peptide chains from unfolding, thus improving the thermal stability of the enzyme [37]. Another study performed on magnetic hNFs embedded with the laccase enzyme showed that hNFs had a significantly better thermal stability than free laccase. One example is that at 55 °C (incubation time of 1 h), the magnetic laccase-hNFs retained 80% of their activity, which was more than that of free laccase. The results showed that a temperature of 35 °C had no effect on the activity, and a temperature of 85 °C made laccase inactive for both laccase-hNFs and free laccase [34].

#### 3.2.2. Storage Stability

The storage stability of enzymes in hNFs is another essential influencing factor that has been intensively studied. The study mentioned above also examined the storage stability of the laccase enzyme in magnetic hNFs. The findings showed that, at room temperature, free laccase lost 77% of its activity after 30 days and 90% after 60 days. On the other hand, laccase-magnetic hNFs sustained 60% of their activity after 30 days and 45% after 60 days. At 4 °C and higher, over 60 days, both laccase-magnetic hNFs and free laccase maintained high activities. The authors suggested that the loss of activity for the free laccase was a result of conformational changes in the enzyme. Additionally, the morphology of the magnetic hNFs was examined using SEM over a 60-day storage period, and there was no visible difference in the nanoflower size and hierarchical structure. In addition, leaching of hNFs at room temperature and at 4 °C over 60 days of storage was studied by examining the protein content in the supernatant of hNFs. The results showed that there was nearly no detectable protein in the supernatant at the two different temperatures, which indicates the stability of the formed hNF complex [34].

Another study synthesized hNFs using copper ions and urease enzyme at different pH values, and examined the enzyme activity after 30 days at 4 °C and room temperature. Both experiments showed that hNFs had a better storage stability over time than the free enzyme. At 4 °C, the hNFs produced at different pH values of 6, 7.4, 8, and 9 lost 22.55%, 3.7%, 10%, and 15% of their initial activities, respectively. On the other hand, free urease lost 73.55% of its initial activity. At room temperature, the hNFs lost 35%, 9.28%, 13.22%, and 22.34% of their initial activities at a pH of 6, 7.4, 8, and 9, respectively, whereas free urease lost 90.25% of its initial activity. These findings not only show that enzyme-embedded hNFs are more stable than their corresponding free enzyme, but also indicate that hNFs synthesized at pH 7.4 have the best storage stability [38].

Nadar et al. prepared hNFs from glucoamylase enzymes and copper ions, and studied their storage stability over 25 days with five-day intervals at 30 °C. Their results showed that the free glucoamylase activity slowly decreased to 68% of its original activity, whereas the hNFs were able to maintain 91% of their original activity [39]. Similarly, Patel et al. found that laccase-hNFs and cross-linked-laccase-hNFs were more stable than free laccase when stored at 4 °C for 60 days. The laccase residual activity was 53.3%, 91.5%, and 3.8% for laccase-hNFs, cross-linked-laccase-hNFs, and free laccase, respectively. Hence, laccase-hNFs and cross-linked-laccase-hNFs had 14 and 24 times, respectively, the free enzyme’s residual activity [30].

### 3.3. Reusability

**Figure 7:**
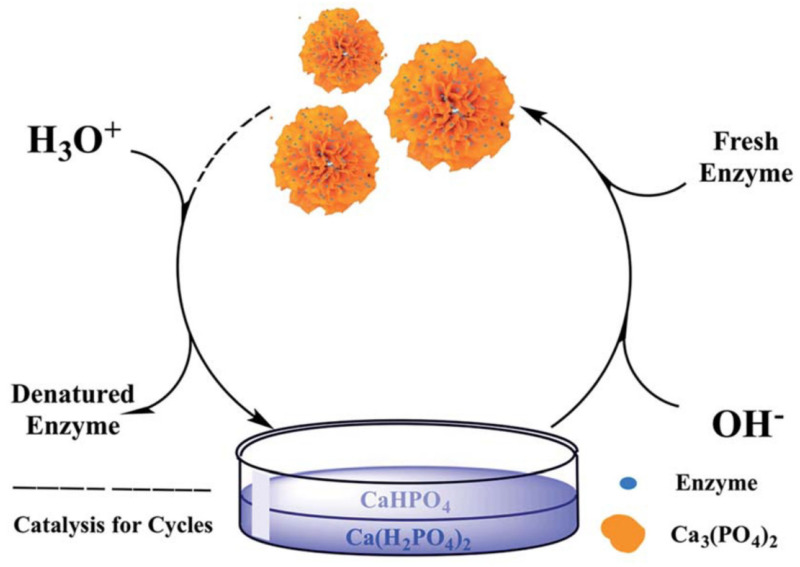
Dual cycle process for enzyme immobilization. Reprinted from [37] with permission from the Royal Society of Chemistry under the Creative Commons Attribution 3.0 Unported Licence.

Research shows that hNFs remain active for multiple reaction cycles before they lose activity regarding reusability. In addition, research has shown that they can be recycled by adding fresh enzymes. Yn et al. rebloomed the hNFs they produced using the following method (Figure 7). First, 0.2 mL of acetic acid or phosphoric acid was added to dissolve the original hNFs. After that, the reaction mixture was heated for 10 min at 100 °C to denature all of the enzymes. The denatured enzymes were then removed via filtration or centrifugation. Then, the solution pH was adjusted to 6.7 using Ca(OH)_2_. A rebloom of the hNFs occurred when fresh enzymes were added to the solution, and co-crystallization occurred with Ca(PO_4_)_2_. The reaction was kept at 4 °C for 24 h. Then, the nanoflowers were separated and used as the original hNFs. The researchers examined both the activity of the dual cycle hNFs and the recovery of Ca(PO_4_)_2_ for six different enzyme models. The results showed no noticeable differences between the original hNFs and the dual-cycle hNFs, which suggested that certain molecules, such as amino acids, do not affect the catalysis of the dual-cycle hybrid nanoflowers. While examining the recovery rate percentage of Ca(PO_4_)_2_ by checking their weight while dry and after recrystallization, the result showed up to a 99% recovery of Ca(PO_4_)_2_ for six enzymes-hNFs. In summary, the activity of the enzymes and the recovery of Ca(PO_4_)_2_ before and after the dual cycle were nearly constant for all of the tested enzyme model hNFs [37].

Memon et al. synthesized hNFs using alcalase and Ca ions. When examining the reusability of the hNFs, the results showed that hNFs maintained 85.4% of their activity during seven cycles [40]. A research group investigated the reusability of hNFs produced from papain enzymes and Zn ions. The findings thus showed that the enzyme could maintain 88.8% of its original activity for ten cycles, but there was a steady decrease in its activity as the cycle number increased [41].

## 4. Types and Synthesis of Organic–Inorganic Hybrid Nanoflowers

As discussed earlier, hybrid nanoflowers are formed between a protein (organic component) and a metal ion (inorganic component). When proteins have a metal-binding site they can form complexes with ions through coordination interactions. For example, enzymes with nitrogen atoms in their amine and amide groups can form complexes with different metals through coordination interactions, i.e., the beginning of hybrid nanoflower synthesis [20,33,42]. Hybrid nanoflowers can be categorized based on the assembly of the particles, the type of protein/enzyme (organic component), and the metal ion (inorganic component) used. Table 1 shows the different metals and proteins used for hybrid nanoflower synthesis. The preparation of different hNFs based on the use of different metals is discussed next.

### 4.1. Copper-Based Hybrid Nanoflowers

**Figure 8:**
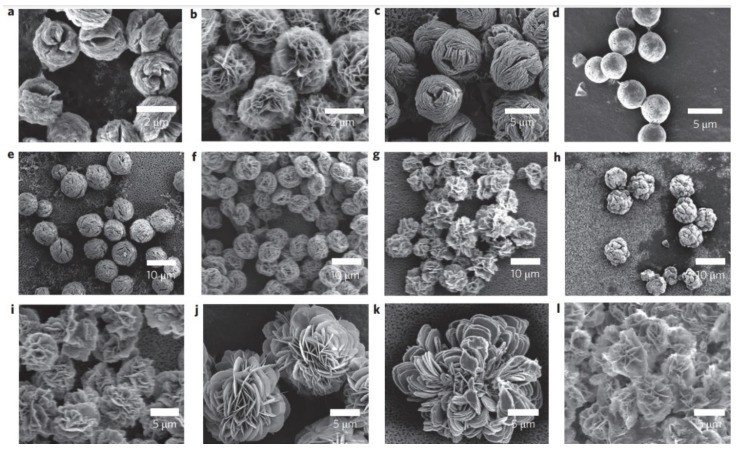
SEM images of hybrid nanoflowers: (**a**–**l**) column 1, a-lactalbumin; column 2, laccase; column 3, carbonic anhydrase; column 4, lipase; at protein. Reprinted from [20] with permission from Springer Nature. Copyright © 2021, Nature Publishing Group. License Number: 5031780508604.

For the first hNFs produced [20], an aqueous CuSO_4_ solution was added to phosphate-buffered saline (PBS) that contained bovine serum albumin (BSA). The reaction succeeded at room temperature and was incubated for 3 days. Scientists then confirmed the synthesis process of hNFs by replacing BSA with other proteins, including α-lactalbumin, carbonic anhydrase, laccase, and lipase (Figure 8). One of the experiments performed on laccase-copper phosphate nanoflowers showed that laccase hNFs had an activity that was 4.5–6.5 higher than that of free laccase for oxidizing catecholamine syringaldazine. Additionally, they showed a good stability and reusability. Another experiment showed that the activity of carbonic anhydrase-embedded hNFs was 2.6-fold higher than the free form activity in the hydration of CO_2_ [20].

**Figure 9:**
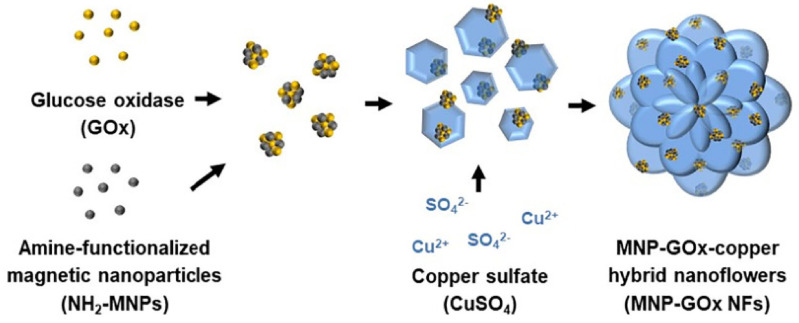
Preparation of MNP-GOx NFs. Reprinted from [111] with permission from Elsevier. Copyright © 2021 Elsevier B.V. License Number: 5031790647430.

As the first discovered hybrid nanoflowers used protein-Cu ions, the majority of work was done on them. Another study showed the synthesis of hNFs from Turkish black radish peroxidase and copper ions. This study showed that hNFs had a better activity and stability in a wide range of pH values, as well as the ability to degrade 90% of Victoria blue dye [43]. As shown in Figure 9, hNFs are formed from glucose oxidase and copper ions embedded in amine-functionalized magnetic nanoparticle-labeled MNP-GOx NFs as antibacterial agents. The results showed that MNP-GOx NFs demonstrated an antibacterial activity with Gram-positive *S.* *aureus* and Gram-negative *E. coli* in a broad spectrum. This was done by disturbing the bacterial cells with the H_2_O_2_ produced by GOx [111]. Yang et al. produced hNFs from copper phosphate and horseradish peroxidase. These hNFs exhibited a linear detection from 100 nM to 100 μM H_2_O_2_. Additionally, they showed a good reusability and excellent storage stability [44]. Sun et al. [112] synthesized copper polyphosphate kinase 2 hNFs and formed an ADP regeneration approach from AMP using hNFs. The resulting hNFs had a better storage stability, in addition to a broader pH and temperature ranges. Additionally, it showed a better ADP production and retained 71.7% of its original activity after ten cycles, which showed good reusability.

**Figure 10:**
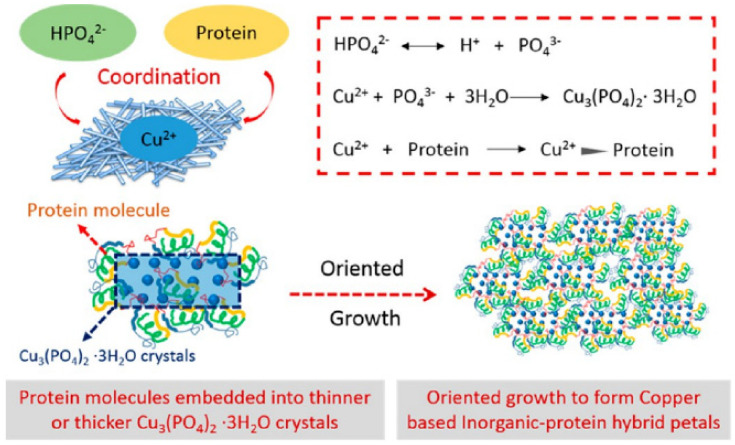
Formation of copper hNFs. Reprinted from [42] with permission from the American Chemical Society. Copyright © 2021 American Chemical Society.

**Figure 11:**
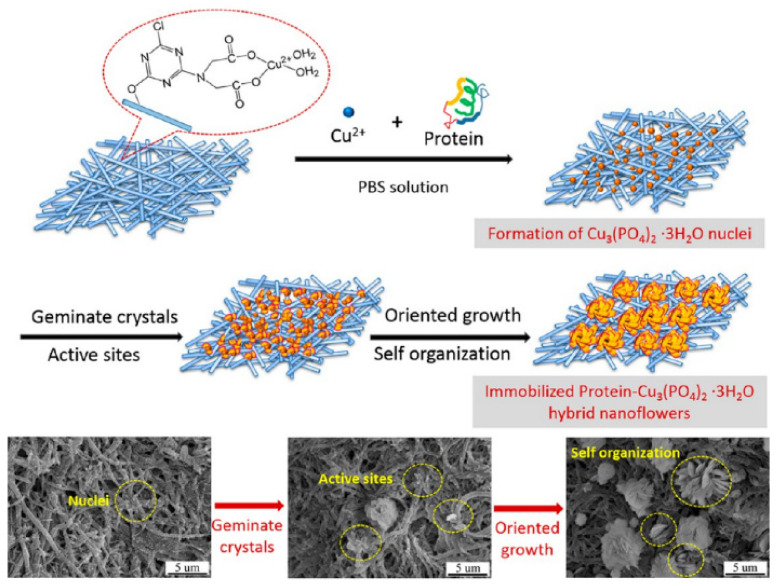
The growing process copper−protein hNFs. Reprinted from [42] with permission from the American Chemical Society. Copyright © 2021 American Chemical Society.

**Figure 12:**
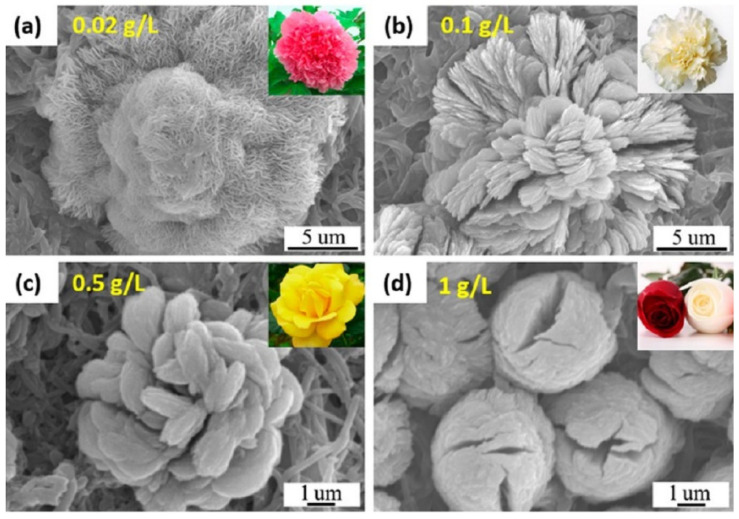
SEM of Cu-BSA hNFs with different protein concentrations, with an incubation time of 6 h. Reprinted from [42] with permission from the American Chemical Society. Copyright © 2021 American Chemical Society.

Li et al. [42] produced hybrid nanoflowers on a nanofiber membrane surface from copper ions and different proteins. This led to the production of a very biocompatible and multilevel surface. The produced copper hybrid nanoflowers displayed an improved stability compared with the free protein that could stem from the protein’s protection from the inorganic crystals. Figure 10 and Figure 11 show the mechanism of how this happens. The gained stability in the hybrid nanoflowers can lead to their introduction to the application, each as biodevices and biocatalysts. The researchers found that by changing the concentration of the protein (Figure 12), the incubation time, the composition of the nanofiber membrane, and the preparation of the mineralizing solutions, the composition and structure of the copper hNFs could be controlled on the nanofiber membrane. The results showed that the different proteins tested (papain, bovine serum albumin (BSA) laccase, and horseradish peroxidase) gave different hNF morphologies, which is supported by previous studies.

### 4.2. Calcium-Based Hybrid Nanoflowers

**Figure 13:**
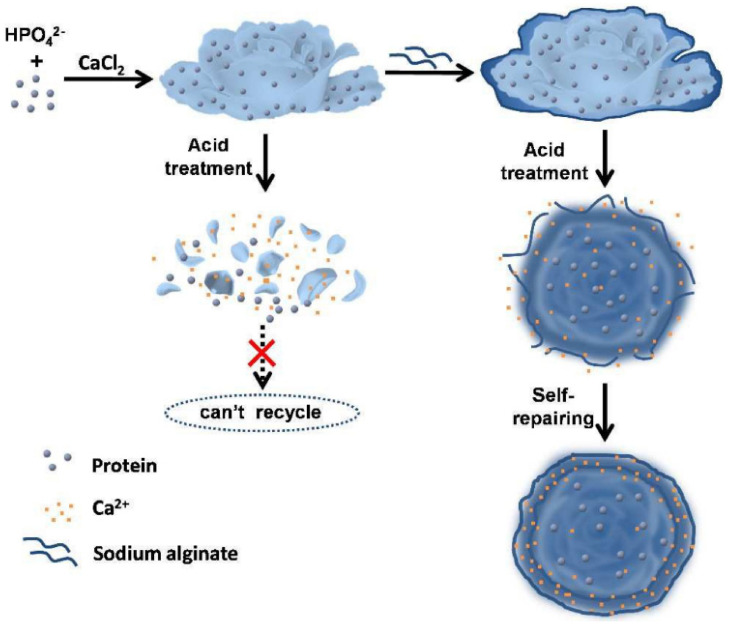
The process of self-repairing sodium alginate (SA)-coated CPO-Ca_3_(PO_4_)_2_ hybrid nanoflowers. Reprinted from [87] with permission from the Royal Society of Chemistry. Copyright © The Royal Society of Chemistry. License Number: 1105129-1.

Even though the most commonly used metal for the synthesis of hybrid nanoflowers is copper, another metal that is heavily used to produce hNFs is calcium. Wang et al. reported the synthesis of α-amylase-CaHPO_4_ hybrid nanomaterials, inspired by the allosteric effect. The work showed three different nanomaterial morphologies: nanoflowers, nanoplates, and parallel hexahedrons. While studying the enzymatic activity of α-amylase in the three different nanomaterial systems developed, and free α-amylase with and without calcium ions, the researchers credited two main factors that increased the enzymatic activity of α-amylase, namely: the allosteric effect of calcium ions with the amine group of the enzyme and the morphology of the nanomaterials [89]. Self-repairing hNFs were built from Ca_3_(PO_4_)_2_ and chloroperoxidase (CPO) with a sodium alginate (SA) coating. The results showed that the immobilized enzyme had similar K_m_ and K_cat_ values, compared with the free enzyme. Additionally, it demonstrated the ability of the immobilized chloroperoxidase to work in acidic conditions, where it was able to maintain more than 85% of its activity after 12 cycles. Figure 13 shows the process of self-repairing SA-coated CPO-Ca_3_(PO_4_)_2_ hybrid nanoflowers [87]. Zhao et al. [92] synthesized calcium hNFs by combining Ca_3_(PO_4_)_2_ and α-acetolactate decarboxylase (ALDC) enzymes. These hNFs had a better activity than the free ALDC.

### 4.3. Manganese-Based Hybrid Nanoflowers

Nearly all of the hybrid nanoflowers produced in the literature use copper or calcium ions. Nevertheless, several studies use different metal ions. One of these metals is manganese; specifically, manganese(II) phosphate is used because of its unique electrochemical properties [113,114,115]. Rai et al. [95] synthesized hNFs using manganese metal as the inorganic component and _L_-arabinose isomerase as the organic component. Recombinant _L_-arabinose isomerase with 474 amino acids was synthesized into E. coli from *Lactobacillus sakai*. Hybrid nanoflowers with a spherical hierarchical morphology were produced using purified recombinant isomerase.

Several studies were performed on the _L_-arabinose isomerase embedded in the hybrid nanoflowers, and a circular dichroism (CD) analysis recorded no change in the isomerase structure. Compared with free _L_-arabinose isomerase, hNF-embedded _L_-arabinose isomerase demonstrated better kinetic parameters. Interestingly, _L_-arabinose isomerase converted approximately 50% of D-galactose to D-tagatose, a rare type of sugar, without adding more manganese to the reaction, showing the possibility of commercial production of this sugar using manganese/_L_-arabinose isomerase hNFs. In addition, these hNFs show good reusability and reproducibility in multiple reaction cycles. Munyeman et al. [96] reported the synthesis of collagen/manganese phosphate hNFs in an environmentally friendly biomineralization method. In this study, collagen was used as the biotemplate agent to produce hNFs. Additionally, it was used to bind the petals of the nanoflowers together. These hNFs had a great catalytic activity in relation to water oxidation. Zhang et al. [97] prepared hNFs from manganese(II) phosphate and bovine serum albumin (BSA). The results showed a good catalytic activity in the fuel cells.

### 4.4. Zinc-Based Hybrid Nanoflowers

Although the synthesis of copper hybrid nanoflowers is simple and easy, its three-day production process is one of its disadvantages. Thus, it is important to reduce the synthesis time to a more appropriate period. This can be done by choosing the right metal ions or by changing the synthetic method. When choosing metal ions, zinc displayed a faster reaction rate towards phosphate radicals than copper ions [35,41,100]. Zhang et al. showed that hNFs were prepared from zinc phosphate Zn_3_(PO_4_)_2_ and lipase. The formation time of Zn(PO_4_)_2_/lipase hNFs took less than three hours, and the formation time of hNFs using Cu(PO_4_)_2_ took three days. Zn(PO_4_)_2_/lipase hNFs showed a great operational stability compared with free enzymes [35]. In another study, hNFs were made using Zn(PO_4_)_2_ and papain, and the resulting hNFs showed a higher activity than free papain, in addition to a better thermal stability and storage life [41]. Zhang et al. [100] prepared hNFs using Zn and bovine serum albumin (BSA) at 25 °C. These hNFs had an average size of 2.3 μm with a surface area of 146.64 cm^2^/g. The hNFs were used for Cu (II) ion adsorption. The adsorption efficiency of the Zn hNFs towards copper ions was 86.33% at 5 min and 98.9% at 30 min. The highest adsorption capacity obtained with these hNFs was 6.85 mg/g. This study showed the ability of Zn(PO_4_)_2_/BSA hNFs to be used as a fast and efficient method for Cu^2+^ removal.

### 4.5. Cobalt-Based Hybrid Nanoflowers

Despite the various studies that investigated the synthesis of hybrid nanoflowers, only a few studies have shown the use of cobalt ions in the formation of hNFs. Kim et al. synthesized protein/cobalt hNFs using BSA and cobalt ions. The work illustrated that the BSA protein could be a template to interact with cobalt phosphate to produce protein-metal hNFs [104]. Kumar et al. [102] produced hNFs using cobalt ions and lipase enzymes. The immobilized lipase showed a 181% higher activity than the free enzyme. Additionally, it offered a better catalytic performance in harsh reaction conditions and higher temperatures.

### 4.6. Iron-Based Hybrid Nanoflowers

Studies that use iron ions to produce hNFs are limited. Ocsoy et al. [107] used Fe^+2^ and horseradish peroxidase (HRP) to produce hNFs. The results showed an approximately 512% increase in the activity of HRP when stored at 4 °C and an approximately 710% increase when stored at room temperature compared with the free enzyme. Additionally, the immobilized HRP lost 2.9% and 10% of its initial activity after 30 days when stored at 4 °C and room temperature, respectively. However, the free HRP lost 68% of its activity when stored at 4 °C and 91% when stored at room temperature.

### 4.7. Multi-Metal-Based Hybrid Nanoflowers

As previously mentioned, there is an extensive range of research and studies in the literature on using different metals to produce hierarchical hybrid nanoflower structures, such as copper, calcium, manganese, zinc, and cobalt. However, few studies have attempted the production of multi-metal-based hybrid nanoflowers; here are a few of them. In 2019, Patel et al. produced hybrid nanoflowers based on copper and zinc ions. The novel multi-metal nanoflowers were synthesized using a laccase enzyme. The Cu/Zn-laccase showed a higher encapsulation yield percentage than the copper-laccase and zinc-laccase hNFs, which were 96.5%, 87.0%, and 90.2%, respectively. The multi-metal nanoflowers (Cu-/Zn-laccase) were 1.2-, 1.5-, and 2.6-fold higher than zinc-laccase, copper-laccase, and free laccase, respectively. Interestingly, the multi-metal nanoflowers showed a charge transfer resistance that was 2.1-fold lower than zinc-laccase hNFs, and when compared with copper-laccase hNFs it was 2.7-fold lower. For the degradation of bisphenol A, the remaining multi-metal nanoflower activity was 1.9-fold higher than that of zinc-laccase hNFs and 5.1-fold higher than that of copper-laccase hNFs [109].

### 4.8. Non-Metal-Based Hybrid Nanoflowers

As mentioned above, there is extensive work in the literature on certain metals, such as copper, calcium, manganese, zinc, cobalt, and iron. Nevertheless, few studies have tried to produce non-metal hybrid nanoflows. In 2018 [110], selenium (non-metal) hybrid nanoflows were synthesized. In recent years, selenium nanoparticles have been studied as drug carriers because they are nontoxic and have a good biological activity and bioavailability [116,117,118,119,120,121]. There have been attempts to produce selenium nanoparticles (SeNPs) with different structures, shapes, and morphologies, including nanoplates, nanotubes, and nanospheres [61,62,63]. The addition of a biopolymer that is functionalized with these nanoparticles has led to an increased stability and control over the shape and size [122,123]. hNFs were synthesized using pullulan/SeNPs, and pullulan was used as a substitute for the use of proteins [110]. SeNPs were then stabilized using folic acid-decorated cationic pullulan (FA-CP), and presented a flower-like structure. The produced nanoflowers showed an excellent drug adsorption for doxorubicin and had a 142.2 mg/g loading capacity. The study showed that doxorubicin’s loading capacity is three times greater in pullulan/SeNP nanoflowers than in spherical SeNPs. Additionally, these hNFs showed a better activity towards cancer cells, and they were less toxic towards normal cells.

### 4.9. Enhancing Hybrid Nanoflowers Synthesis

One of the most significant drawbacks of hNFs is their size, which is usually in the nano- or micro-scale range. Thus, it is difficult to separate them from their reaction mixture. Some studies have been performed to improve the synthetic processes of hybrid nanoflowers. Recently, scientists developed supported hybrid nanoflower methods to overcome some of the drawbacks of the use of hNFs. Alginate gel beads were used to entrap α-acetolactate decarboxylase/calcium hNFs. These entrapped hNFs showed a better stability and recyclability than the free enzyme [92]. Another study by Zhu et al. used a cellulose acetate membrane for laccase/copper ion hNFs. The captured hNFs showed a high reproducibility and reusability for phenol detection [55]. Cao et al. [124] produced a glassy carbon electrode (GCE) surface on which bovine serum albumin (BSA)/Ag nanoflowers were immobilized, coupled with a targeting lectin molecule for detecting human colon cancer cells. This sensor showed specificity for a cell expressing sialic acid. Therefore, it has a possible application for monitoring tumor cells.

### 4.10. Morphology of Hybrid Nanoflowers

hNFs commonly have a hierarchical structure and nanoplate petals that look like flower petals. These nanoplates/nanopetals are made of enzyme and metal phosphate. From the first hybrid nanoflower produced by J. Ge et al. in 2012, there have been numerous synthesis techniques to produce hNFs, which have resulted in different hNFs with different microstructures [125,126,127]. Table 2 shows the most common hNF microstructure morphologies, which are spherical, rosette, and rhombic, with their equivalent flowers in nature. Typically, hNFs have a diameter range between 1–30 µm. There are many factors and reaction conditions that control the shape and size of hNFs; these factors include (I) the type of enzyme used; (II) the type of metal ion; and (III) the reaction condition, which includes the pH value, the reaction temperature, and time [69]. Table 3 shows how morphology differs with changing the factors and conditions.

### 4.11. The Type of Enzyme Used

As mentioned above, for hNFs to form, the presence of an enzyme is essential. Enzymes attach to the petals of various metal phosphate nanoplates. Numerous studies have shown that different enzymes lead to the formation of different hNF morphologies [21,22,23,24]. Chung et al. [52] developed hNFs using copper ions with various enzymes, including glucose oxidase (GOx), laccase, and catalase. Each enzyme gave a unique microstructure morphology of the hNFs, as illustrated in Table 3 (1, 2, and 3).

Zhang et al. [35] showed the use of lipase enzyme to form hNFs (Table 3 (4)), which has a completely different morphology from the three previously mentioned enzymes of glucose oxidase (GOx), laccase, and catalase. Another study used enzymes, laccase, papain, and horseradish peroxidase (HRP) to synthesize different shapes and sizes of hNFs [42]. Research explains that the diversity of hNF morphology arises from the various amide groups on the surface of the enzyme used. Thus, it presents different nucleation sites with varied geometries and densities for phosphate metal nanoplates to bind [20,33]. In addition, Lin et al. revealed that the employed enzyme’s molecular weight has an impact on the hNF structure [36].

Additionally, it has been determined that the concentration of the enzyme plays a role in the final morphology of hNFs, where it can affect the size and density of the nanoplates [56]. Several studies have indicated that the lower the enzyme concentration used, the larger the size of the hNFs formed with a lightly constructed structure. In addition, an increase in the employed enzyme content leads to a denser packed structure, although the size will decrease if the enzyme concentration reaches a particular value [72,86,99]. The reason behind this is the subsequent increase in the number of nucleation sites [68,103]. Table 3 (5–12) shows the synthesis of two different hybrid nanoflowers using two different enzymes (lipase and papain) with the same metal ion (Zn). As seen from the table, the morphology of both hNFs dramatically changes with an increased amount of enzyme, from a rhombus shape to a square, oval shape. With the continuous addition of the enzyme (an excess amount), the shape changes into a dense cluster structure with cracks instead of a well-formed flower shape [35,41].

### 4.12. The Type of Metal Ion Used

In the formation of hNFs, metal ions play an essential role in the primary crystal nucleation step and metal-enzyme coordination to produce hNFs. Table 3 (13–15) shows a study where hNFs were synthesized using a chloroperoxidase enzyme with three different metals (copper, cadmium, and cobalt). It was observed that copper and cobalt hNFs had similar morphologies, while cadmium hNFs were completely different. Copper and cobalt hNFs had a spherical flower-like structure, whereas cadmium hNFs had a butterfly-like structure [29]. The effect of the metal ion concentration on the morphology of hNFs was also studied. The results show that with an increase in the metal ion concentration, the hNF morphology becomes much denser [38,85].

Rai et al. [95] produced hNFs using _L_-arabinose isomerase as the organic component, and 13 metal ions, including manganese, cobalt, magnesium calcium, potassium, sodium, nickel, and lithium, as the inorganic component. It was determined that manganese and cobalt ions can substantially improve the catalytic activity of the enzyme. In contrast, the magnesium calcium, potassium, sodium, nickel, and lithium ions had no impact on the enzyme activity.

### 4.13. Reaction Condition (pH, Temperature, and Time)

It is well known that the charge of the enzyme varies at different pH values. Thus, leading to the enzyme’s different interaction capabilities in the formation of hNFs will influence the morphology of the hNFs [74]. Nadar et al. [39] examined the effect of changing the pH on hNFs synthesized using copper ions and glucoamylase enzymes. The study tested the pH range from 3.5 to 9.5. The net change in free glucoamylase enzymes is neutral, and it has an isoelectric point (pI) of approximately 6. The enzyme is expected to have a positive charge below the isoelectric point, and a negative charge above the isoelectric threshold. No nanoflower formation was observed at pH values of 3.5, 4.5, and 5.5 (below the isoelectric point). This can be explained by the fact that the positively charged protonated glucoamylase enzyme had an extremely strong repulsion with the positive copper metal ions at a low pH. Hence, no nanoflowers were formed. At pH values higher than the pI point, the charge on the glucoamylase enzyme surface was negative because of deprotonation. Glucoamylase/Cu^2+^ ions hNFs were formed in this pH range. It was observed that at pH 7.5 the nanoflowers were less packed, which is attributed to the small rise in the negative charge on the enzyme. At pH 9.5, nanoflowers did not form. This is because of the increased repulsion between the negative charges that are highly dense on the surface of the glucoamylase enzyme. Therefore, Cu_3_(PO_4_)_2_·3H_2_O petals repel one another rather than attaching. Table 3 (16–19) shows the morphology of the synthesized lipase/copper hNFs at different pH values, where the enzyme concentration is 1.0 mg/mL. The pH values of phosphate-buffered saline (PBS) were adjusted to 6.0, 7.4, 8.0, and 9.0. It was noted that the density of the petals decreased as the pH value increased. However, the diameters and the size of the formed nanoflowers remained the same [69].

Another important reaction condition that affects the morphology of hNFs is temperature. Temperature can play a vital role by initiating the various diffusion activities of the enzyme at different applied temperatures. Thus, nanoflowers have different degrees of density in petals and alter their size and diameter [43]. Table 3 (20–23) shows the effect of temperature on the production of calcium ions/elastin-like polypeptide (ELP) hNf and copper ions/ELP hNFs. ELPs are a class of polypeptides derived from an amino acid sequence of naturally accusing elastin in humans. Their pentapeptide sequence is Val-Pro-Gly-X-Gly, where X is any amino acid, other than from Pro. ELPs have a key transition temperature (T_t_), where ELPs are soluble in the solution below it. Nevertheless, above this temperature, ELPs will suffer a phase transition that is destructive to the polypeptide. As seen from the table, the morphology of the hNFs synthesized below T_t_ (4 °C) had larger, more expanded petals. However, the hNFs synthesized above T_t_ (37 °C) had a more closed structure [93]. Another study, shown in Table 3 (24–27), demonstrated that hNFs formed at 20 °C had a more oval spherical shape with cracks. However, when the temperature increased above 40 °C, the shape of the hNFs changed to a more sheet-like structure, and as the temperature further increased, the hNF sheet morphology increased [35]. Altinkaynak et al. [51] showed the production of hNFs from copper ions and lactoperoxidase (LPO), and it was concluded that at a lower temperature, the petals of the hNFs became more compacted. This result is shown in Table 3 (28–29). For this, the ideal temperature for the synthesis of the best hNF structure must be investigated.

An additional factor that can affect the hNF morphology is the reaction time. The reaction time of the hNFs depends on the method used to produce them. Since the discovery of hNFs, there have been different time intervals used when producing them. Some of these time intervals include 3 days [20,30,38,44,68,111], 24 h [34,39,109], 3 h [35], and 5 min [52,58]. Table 3 (30–32) shows the different stages of hNF formation. The first step, nucleation, which is the formation of primary copper phosphate crystals, occurs between 0 and 2 h. The second step, growth, is when the metal ion and the enzyme form large agglomerates, which are the primary petals. This occurs between 2 to 8 h. In the last step, complete hNFs are formed, which occurs between 8 to 24 h [39].

## 5. Applications for Hybrid Nanoflowers

**Figure 14:**
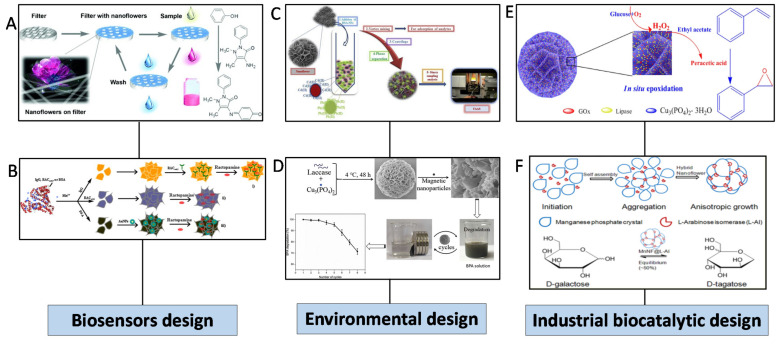
Summary of different applications used for hNFs. (**A**) Fast detection of phenol using laccase/cooper phosphate hNFs integrated into a membrane. Reprinted from [55] with permission from John Wiley and Sons. Copyright © 2021 WILEY-VCH Verlag GmbH and Co. KGaA, Weinheim. License Number: 5031800087304. (**B**) Detection of ractopamine using a protein/manganese ion hNF electrochemical biosensor. Reprinted from [98] with permission from Elsevier. Copyright © 2021 Elsevier B.V. License Number: 5031800271904. (**C**) Selective separation of cadmium and lead in water, cigarette, and hair samples using BSA/copper ions hNFs. Reprinted from [131] with permission from Elsevier. Copyright © 2021 Elsevier B.V. License Number: 5031800434687. (**D**) Degradation of bisphenol A using laccase/cooper phosphate hNFs. Reprinted from [34] with permission from Elsevier. Copyright © 2021 Elsevier B.V. License Number: 5031800600650. (**E**) Epoxidation of alkenes using dual enzyme system GOx and lipase/copper ions hNFs. Reprinted from [77] with permission from Elsevier. Copyright © 2021 Elsevier Ltd. License Number: 5031800753597. (**F**) Transformation of D-Galactose to D-Tagatose using L-Arabinose Isomerase/manganese ions hNFs. Reprinted from [95] with permission from the American Chemical Society. Copyright © 2021 American Chemical Society.

The excellent catalytic properties of hNFs have provided a wide selection of applications. Some of these applications are in the fields of biosensors, biomedical, bioremediation, and industrial biocatalysts [45,128,129,130]. Different examples of these applications are illustrated in Figure 14.

### 5.1. Biosensors

A biosensor is an analytical device employed to detect chemical substances and has recognition elements (biological components), such as enzymes, antibodies, microorganisms, and DNA [132,133]. There is a higher demand for cheap, easy, fast, and sensitive analytical biosensors [134]. Synthesized organic–inorganic hybrid nanoflowers have recently been used as novel biosensors with different classes of enzymes and metal ions because of their high surface area and sensitivity [135].

Zhu et al. produced hNFs for the fast detection of aqueous phenol using laccase enzyme and copper phosphate integrated into a membrane. The results showed the ability for the rapid on-site detection of phenol in water [55]. Zhang et al. reported the production of an electrochemical biosensor using Mn ions/protein hNFs. These hNFs were able to detect ractopamine and showed a high activity and an excellent electrochemical performance [98]. Additionally, copper/HRP hNFs could be used as a sensor for dopamine detection [31]. Sun et al. [80] used dual enzyme (HRP + GOx) hNFs as colorimetric sensors for glucose detection.

### 5.2. Environmental Applications

Environmental pollutants are a rising concern worldwide. Multiple methods have been used to eliminate these pollutants in the environment. One of these methods is bioremediation, which utilizes microorganisms and enzymes to reduce the concentrations of these harmful pollutants to an acceptable range [136,137,138,139]. Although enzymatic treatment has been shown to be effective, it faces some challenges, including a low activity, stability, and sustainability. Recently efforts have been made to immobilize enzymes on different support materials that can eliminate some of the challenges of using free enzymes [140,141]. However, traditional immobilization techniques can lead to a reduced activity of the enzyme compared with the free enzyme. Thus, enzyme immobilization of hNFs has been shown to increase the activity, stability, and reusability of the enzyme [24,37]. Yilmaz et al. produced hNFs from BSA/copper ions for the selective separation of cadmium (Cd) and lead (Pb) in water, cigarette, and hair samples. The method reported a low detection limit for Cd and Pb compared with the other existing methods [131]. The Rong group synthesized hNFs using laccase/copper ions and loaded them onto a treated Cu foil surface. These hNFs showed a high decolorization efficiency and rate on Congo red (CR) dye compared with the free enzyme. In addition, the hNFs showed a good stability and reusability [32]. Fu et al. [34] produced enzyme hNFs that could degrade 100% of bisphenol A within 5 min.

### 5.3. Industrial Biocatalytic Applications

As a result of the great activity, stability, and reusability of enzymes immobilized on hNFs, there has been increasing interest in their use in industrial biocatalytic applications, making them a prominent research area [142]. Zhang et al. [77] used hNFs made from a dual enzyme system GOx + lipase/copper ions for the epoxidation of alkenes. The hNFs showed an excellent reusability, retaining 82% of their activity after ten reaction cycles. First, hydrogen peroxide is produced by GOx from glucose and is then directly used by lipase. As a consequence of lipase, carboxylic ester is converted into peracid. After that, peracid is used in the epoxidation of alkenes.

Hybrid nanoflowers are also used in the food manufacturing industry [62,95]. Bai et al. and Xu et al. were able to synthesize two expensive rare sugars: L-ribulose and D-tagatose. These sugars are monosaccharides, but are very difficult to find in nature. Multiple chemical reactions have been employed to manufacture L-ribulose and D-tagatose from disaccharides and polysaccharides. They used L-arabinose isomerase as the organic component in hNFs and manganese ions [95] and copper ions [62] to produce sugars. The conviction rates were 50% and 61.88% for the manganese hNFs and copper hNFs, respectively. In addition, hNFs can be used in the brewing industry. hNFs made from α-acetolactate decarboxylase (ALDC)/calcium ions can be used to inhibit diacetyl formation in beer, which results in a buttery off-flavor [92].

## 6. Cost Assessment and Industrial Integration Aspects

As discussed above in the representative sections/subsections, the development of immobilized enzyme-based biocatalysts using different nanoscale materials, including hybrid nanoflowers (hNFs), has gained remarkable interest. The rationale behind this rising research trend in immobilized enzyme-based biocatalysts is the unique structural, physicochemical, and functional promises that enzyme-loaded hNFs offer with industrial integration potentialities. However, early-stage cost assessment and market analysis of enzyme-loaded biocatalytic nano-constructs, both pristine and hybrid, are equally essential to govern their industrial appropriateness [143,144]. The overall cost of the enzyme-based product and market analysis can be made via life-cycle assessments (LCAs) and/or techno-economic scrutinizes as powerful tools. By taking the added value of these tools, an array of immobilized enzyme-based biocatalysts have been or are being assessed for commercial-scale biocatalytic processes [145,146]. More specifically, from the cost and sustainability considerations, LCAs provide deep insight into the material, energy consumption, wasteful protection, and deprotection points in order to ensure the sustainability of the entire industrial process. The cost-effective ratio related to the energy consumption, chemical inputs, wasteful protection, and deprotection steps involved in the traditional industrial processes can be controlled effectively by implementing immobilized enzyme-based biocatalysts, e.g., enzyme-loaded hNFs, which are recoverable and reusable. For example, in several industrial processes, a massive amount of heat energy is required in the form of steam to preheat the feedstock for treatment purposes. In contrast, this can be significantly reduced by alternating the process with immobilized enzyme-driven treatment [144]. According to the Global Industrial Enzymes Market Size and Regional Forecasts 2020–2027, in 2019, the global enzymes market was worth $8636.8 Million USD, which is projected to reach up to $14,507.6 Million USD with a compound annual growth rate of 6.5% over the duration of 2020–2027 [147]. Conclusively, the large-scale industrial integration and/or scale-up deployment of immobilized enzyme-based biocatalysts technology requires a proper understanding of both technological and economic aspects, as well as a good perception of the larger market forces.

## 7. Conclusions and Future Perspectives

Since the discovery of organic–inorganic hybrid nanoflowers in 2012, the topic has become a prominent research area and numerous types of biomolecules/inorganic hybrid nanoflowers have been explored. These hybrid nanoflowers have recently shown their ability to become stable, easy, fast, efficient, and recyclable for different biomolecules, specifically enzymes immobilizing host platforms. Additionally, hybrid nanoflower applications have been extended to biosensor designs, environmental treatment, bioassays, and different industrial biocatalysis. Studies have shown that hybrid nanoflowers lead to an enhancement in the immobilized enzyme’s catalytic activity, resulting from the higher surface area of hNFs, a reduced mass transfer limitation, and favorable enzyme conformation in hNFs. Another potential advantage is that in some cases, enzymes immobilized on hybrid nanoflowers behaved better than other immobilization platforms, especially in terms of their reusability. For example, lipase enzyme immobilized on hybrid nanoflowers showed an excellent operation stability as they retained up to 94.5% of their activity, even after eight cycles of reaction [35]. Similar results were obtained by Li et al., where they could efficiently use the lipase/calcium nanoflowers up to six times [148]. On the other hand, when lipase was immobilized on the Fe_3_O_4_@MIL-100(Fe) composite it lost around 15% of its activity after only five cycles [149]. Similarly, when the alcalase enzyme was immobilized on hybrid nanoflowers [40] and glyoxyl supports [150], the nanoflowers could be recycled efficiently up to seven times, as opposed to the agarose beads (only five times). In addition, HRP immobilized on NH_2_-modified magnetic Fe_3_O_4_/SiO_2_ particles was only able to do four cycles before losing most of its activity [151], whereas HRP immobilized on hybrid nanoflowers only lost 25% of its activity after six reaction cycles [49].

Although there has been a rapid increase in the use of hybrid nanoflowers in recent years, there are still some key challenges that need to be addressed. First, the interaction between the organic component (enzymes) and the inorganic component (metal ions) needs to be examined more intensively, providing more information about the hybrid nanoflower design with a well-retained biological activity. This will help with controlling the morphology as well as adjusting the properties of the synthesized hybrid nanoflowers. In addition, this would facilitate the production of new kinds of hybrid nanoflowers for a particular application purpose. In addition, the nucleation step in the inorganic phase is still not described quantitatively in the presence of different amounts of enzymes. Second, hybrid nanoflowers with multiple enzymes or dual enzyme systems have not been thoroughly investigated. Third, more research needs to be done for examining the production of hNFs in organic media, as most of the synthesized hNFs are in aqueous media. This would be beneficial for industrial biocatalysis applications. Finally, the industrial application of hybrid nanoflowers can expand to include energy application like fuel cell fabrication and biodiesel. In summary, we believe that organic–inorganic hybrid nanoflower research will expand the application arena of hNFs and lead to smart solutions for modern problems.

## Figures and Tables

**Table 1:** Different hNFs based on the different metal ions and enzymes used.

Metal Ion	Enzyme	Class of Enzyme	Application	Reference
Copper (II) ions	Turkish black radish	Peroxidase	Dye decolorization	[43]
	Horseradish peroxidase	Peroxidase	Detection of hydrogen peroxide	[44]
	Horseradish peroxidase	Peroxidase	Detection of *E. coli*	[45]
	Horseradish peroxidase	Peroxidase	Detection of hydrogen peroxide and phenol	[36]
	Horseradish peroxidase	Peroxidase	Detection of amyloid	[46]
	Horseradish peroxidase	Peroxidase	-	[47]
	Horseradish peroxidase	Peroxidase	-	[42]
	Horseradish peroxidase	Peroxidase	-	[48]
	Horseradish peroxidase	Peroxidase	-	[49]
	Horseradish peroxidase	Peroxidase	-	[50]
	Chloroperoxidase (CPO)	Peroxidase	Dye decolorization	[29]
	Soybean peroxidase (SBP)	Peroxidase	-	[24]
	Lactoperoxidase (LPO)	Peroxidase	-	[51]
	Catalase	Peroxidase	Glucose biofuel cell	[52]
	Catalase	Peroxidase	Detection of hydrogen peroxide	[53]
	Catalase	Peroxidase	-	[50]
	Laccase	Laccase		[42]
	Laccase	Laccase	Degradation of the pollutant bisphenol A	[34]
	Laccase	Laccase	Decolorization of Congo Red (CR)	[32]
	Laccase	Laccase	Dye decolorization	[30]
	Laccase	Laccase	-	[54]
	Laccase	Laccase	Detection of phenol	[55]
	Laccase	Laccase	Synthesis of viniferin	[56]
	Laccase	Laccase	Synthesis of viniferin	[57]
	Laccase	Laccase	Glucose biofuel cell	[52]
	Glucose oxidase (GOx)	Carbohydrase	Detection of glucose	[58]
	Glucose oxidase (GOx)	Carbohydrase	-	[50]
	Glucose oxidase (GOx)	Carbohydrase	Glucose biofuel cell	[52]
	Glucose oxidase (GOx)	Carbohydrase	-	[59]
	_L_-Xylanase	Carbohydrase	-	[60]
	Glucoamylase	Carbohydrase	-	[39]
	α-Glycosidase	Carbohydrase	Testing for α-glycosidase inhibitors	[61]
	L-Arabinose Isomerase	Carbohydrase	Preparation of two expensive rare sugar L-ribulose and D-tagatose	[62]
	Candida rugosa lipase	Lipase	-	[63]
	Candida antarctica lipase	Lipase	Epoxidation of fatty acids	[64]
	pseudomonas cepacia lipase	Lipase	-	[65]
	Bacillus subtilis lipase	Lipase	Transesterification of (R,S)-2-pentanol	[66]
	Lipase	Lipase	p-nitrophenol butyrate hydrolysis	[67]
	Lipase	Lipase	-	[27]
	Lipase	Lipase	-	[68]
	Lipase	Lipase	-	[69]
	Lipase	Lipase	Uses as green media solvent	[70]
	Lipase	Lipase	Biodiesel synthesis	[71]
	proteinase K	Protease	Detergent additive	[72]
	Alkaline protease	Protease	-	[73]
	Trypsin	Protease	Protein digestion	[23]
	Papain	Protease	-	[42]
	Papain	Protease	-	[74]
	Papain	Protease	-	[69]
	Papain	Protease	-	[75]
	Cholesterol oxidase (ChOx) and horseradish peroxidase	Dual enzyme	Detection of cholesterol	[76]
	Glucose oxidase and lipase	Dual enzyme	Epoxidation of alkenes	[77]
	Acetylcholinesterase and choline oxidase	Dual enzyme	On-site detection of the pesticide organophosphorus	[78]
	Glucose oxidase and horseradish peroxidase	Dual enzyme	Monitoring urinary tract infection (UTI) in clinical practice	[79]
	Glucose oxidase and horseradish peroxidase	Dual enzyme	Glucose sensor	[80]
	Glucose oxidase and horseradish peroxidase	Dual enzyme	Detection of glucose	[81]
	Glucose oxidase and horseradish peroxidase	Dual enzyme	Detection of glucose	[82]
	Cytochrome P450	Others	Oxidation of sulfides	[83]
	_L_-Arabinitol 4-dehydrogenase	Others	_L_-xylulose production	[84]
	Urease	Others	-	[38]
	Brevibacterium cholesterol oxidase (COD)	Others	-	[85]
	Carbonic anhydrase	Others	-	[26]
	2,4-dichlorophenol hydroxylase	Others	-	[86]
Calcium (II) ions	chloroperoxidase (CPO)	Peroxidase	-	[87]
	Chitosan and Catalase	Peroxidase	-	[88]
	α-amylase	Carbohydrase	-	[89]
	β-Galactosidase	Carbohydrase	Protein biomarker	[90]
	Candida antarctica lipase	Lipase	-	[37]
	Porcine pancreas lipase	Lipase	-	[37]
	Thermomyces lanuginosus lipase	Lipase	-	[37]
	Burkholderia cepacia lipase (BCL)	Lipase	-	[21]
	Alcalase	Protease	-	[40]
	Bromelain	Protease	-	[37]
	Trypsin	Protease	-	[37]
	Papain	Protease	-	[37]
	α-chymotrypsin	Protease	Digestion of bovine serum albumin (BSA) and human serum albumin (HSA)	[22]
	Dual enzyme: Aldehyde ketone reductase and alcohol dehydrogenase	Dual enzyme	Production of (S)-1-(2, 6-dichloro-3-fluorophenyl) ethyl alcohol, a key chiral alcohol that is an intermediate of Crizotinib, an anti-cancer drug	[91]
	α-Acetolactate decarboxylase (ALDC)	Others	Inhibition of diacetyl formation in beer	[92]
	Elastin-like polypeptide (ELPs)	Others	Detection of H_2_O_2_	[93]
	Invertase	Others	*E. coli* detection from milk	[94]
	Carbonic Anhydrase	Others	-	[26]
Manganese (II) ions	L-Arabinose Isomerase	Carbohydrase	Transformation of D-Galactose to D-Tagatose	[95]
	Collagen	Others	Water oxidation	[96]
	Bovine serum albumin (BSA)	Others	Catalysis in fuel cells	[97]
	Carbonic Anhydrase	Others	-	[26]
	Ractopamine antibody	Others	Electrochemical biosensors ractopamine detection	[98]
Zinc (II) ions	Lipase	Lipase	Regioselective acylation of arbutin	[99]
	Lipase	Lipase	-	[35]
	Papain	Protease	-	[41]
	Bovine serum albumin (BSA)	Others	Adsorption of heavy metal ions	[100]
Cobalt (II) ions	Chloroperoxidase (CPO)	Peroxidase	Dye decolorization	[29]
	_D_-Psicose 3-Epimerase (DPEase)	Carbohydrase	-	[101]
	Lipase	Lipase	-	[102]
	ω-Transaminase	Others	-	[103]
	Bovine serum albumin (BSA)	Others	-	[104]
	Bovine serum albumin (BSA)	Others	-	[105]
	His-tagged enzyme	Others	Redox reaction cycles	[106]
Iron (II) ions	Horseradish peroxidase	Peroxidase	-	[107]
	Glucose oxidase (GOx)	Carbohydrase	-	[108]
Multi-metal (Copper+ Zinc)	Laccase	Laccase	Degradation of the pollutant bisphenol A	[109]
Non-metal (Selenium)	Pullulan (polysaccharide polymer)	Others	-	[110]

**Table 2:** Characteristic of hybrid nanoflowers.

Nature Flower	Shape	Example		SEM Image	Size	Reference
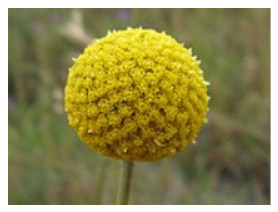	Spherical	The enzyme: *Brevibacterium* cholesterol oxidase (COD)	The metal: copper	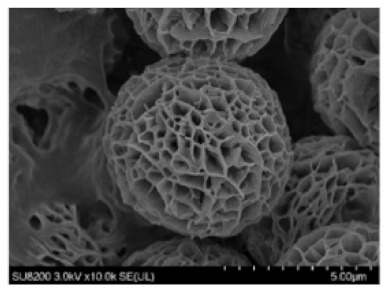	5 μm	[85]
		The enzyme: laccase	The metal: copper	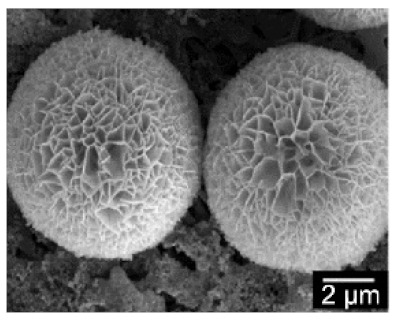	1 min sonication: ~2 µm 5 min sonication: ~8 µm 7 min sonication: No additional increase in the size	[54]
		The enzyme: glucose oxidase (GOx) + horseradish peroxidase (HRP)	The metal: copper	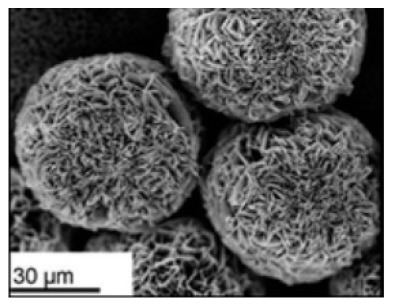	30 μm	[80]
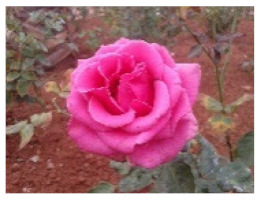	Rosette	The enzyme: α-glycosidase	The metal: copper	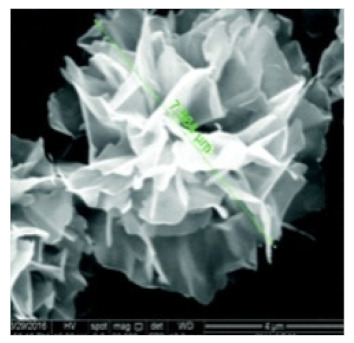	7.564 μm	[61]
		The enzyme: catalase	The metal: copper	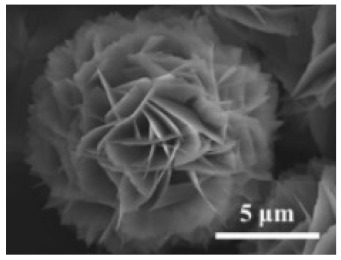	10 to 20 μm	[53]
		The enzyme: streptavidin + horseradish peroxidase (HRP)	The metal: copper	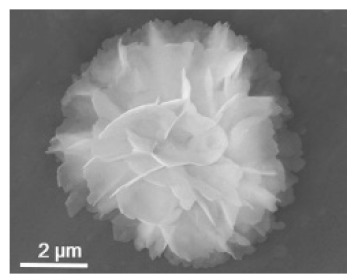	5 μm	[49]
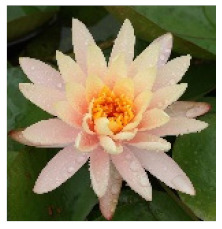	Rhombic	The enzyme: D-psicose 3-epimerase (DPEase)	The metal: cobalt	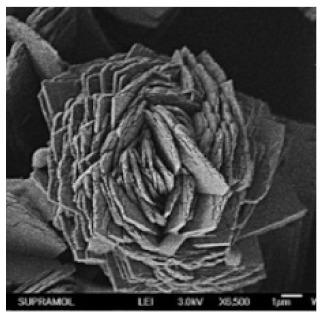	7 µm	[101]
		The enzyme: papain	The metal: zinc	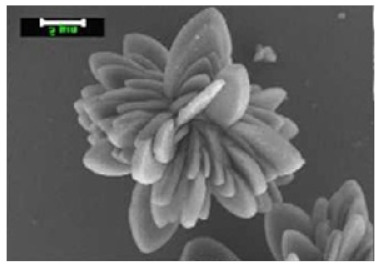	-	[41]
		The enzyme: laccase	The metal: copper	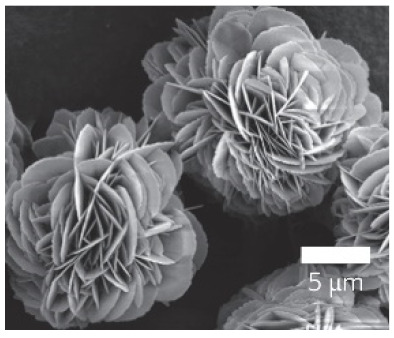	-	[20]

**Table 3:** Different factors and conditions that affect the morphology of hNFs.

Enzyme	Morphology				Reference
Type of enzyme used	1. Enzyme: Glucose oxidase (GOx) 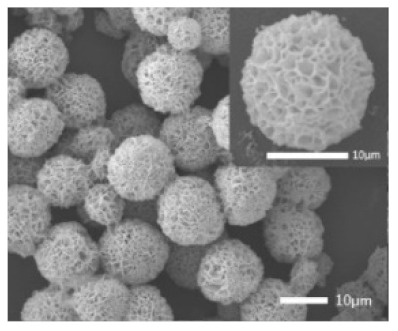	2. Enzyme: Laccase 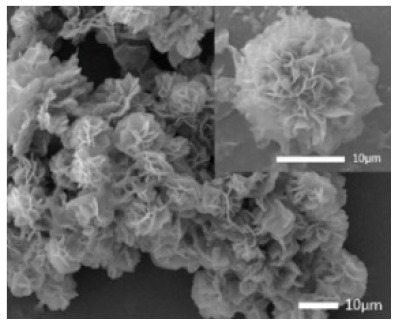	3. Enzyme: Catalase 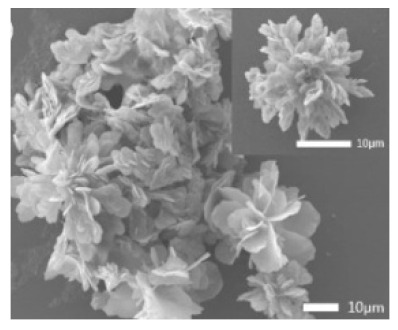	4. Enzyme: Lipase 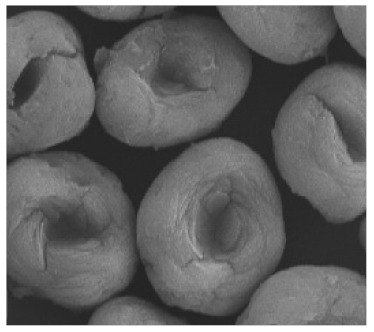	[35,52]
Different amount of the enzyme # Example 1	5. 0.01 g Lipase 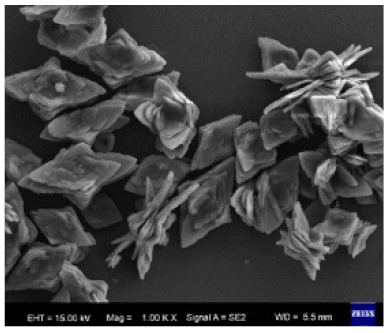	6. 0.025 g Lipase 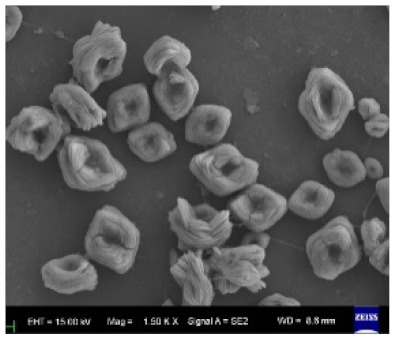	7. 0.05 g Lipase 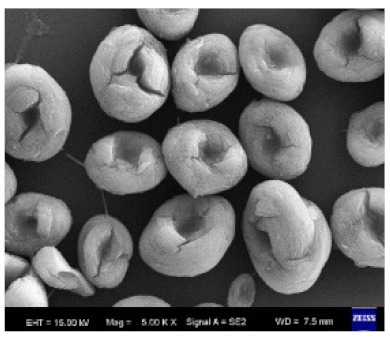	8. 0.10 g Lipase 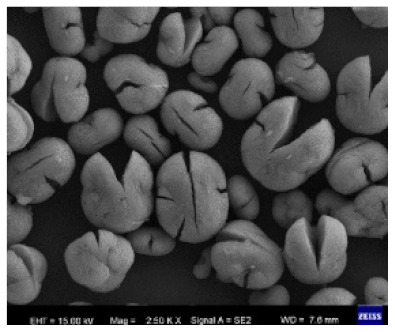	[35]
Different amount of the enzyme # Example 2	9. 0.025 g papain 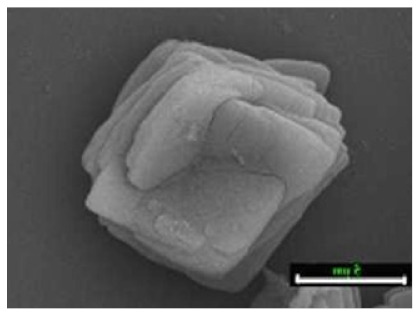	10. 0.05 g papain 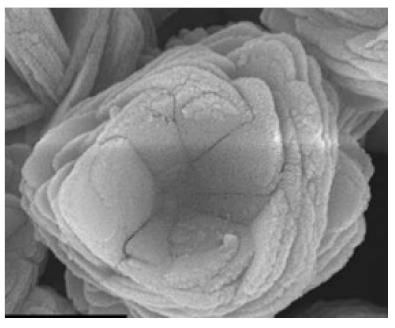	11. 0.1 g papain 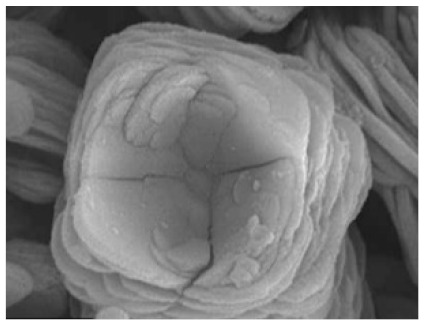	12. 0.25 g papain 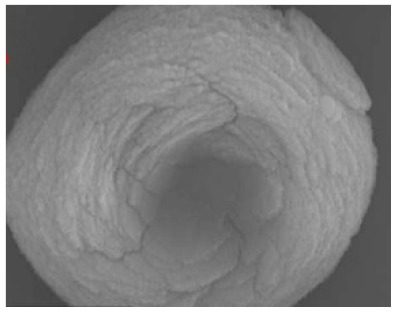	[41]
Metal ion	Morphology				
Type of metal ion used	13. Copper 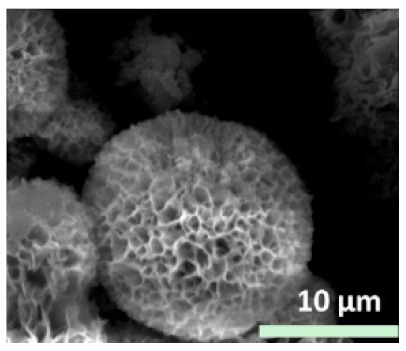	14. Cadmium 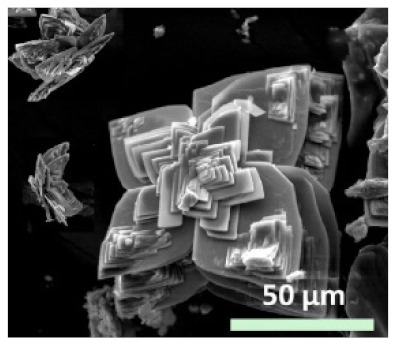	15. Cobalt 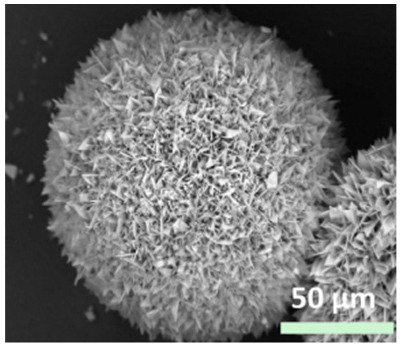	-	[29]
Reaction pH	Morphology				
Different pH values	16. pH 6 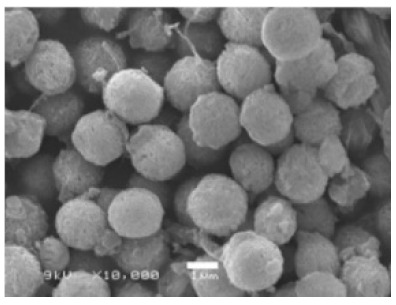	17. pH 7.4 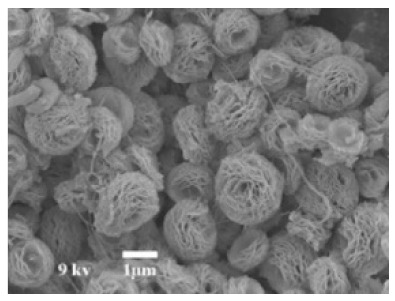	18. pH 8 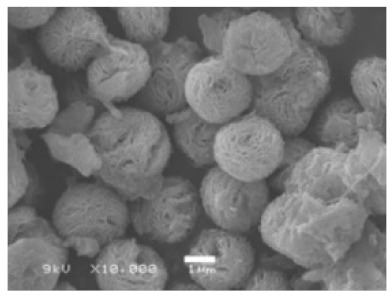	19. pH 9 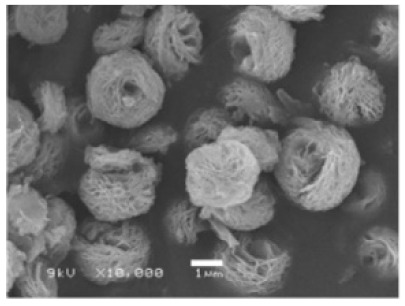	[69]
Reaction temperature	Morphology				
Different reaction temperature # Example 1	20. Temperature: Below T_t_, at 4 °C ELP/**Ca** hNFs 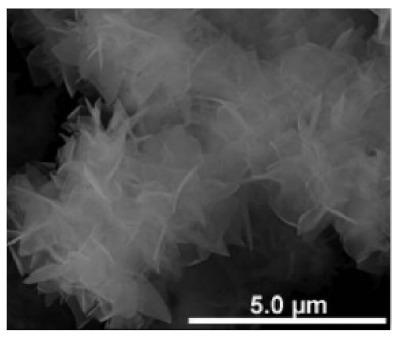	21. Temperature: Above T_t_, at 37 °C ELP/**Ca** hNFs 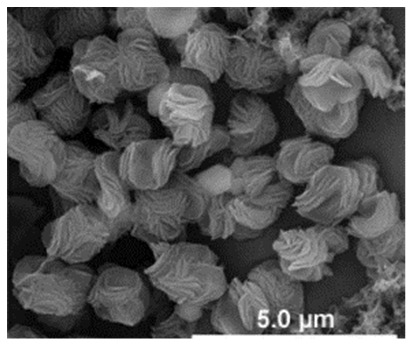	22. Temperature: Below T_t_, at 4 °C ELP/**Cu** hNFs 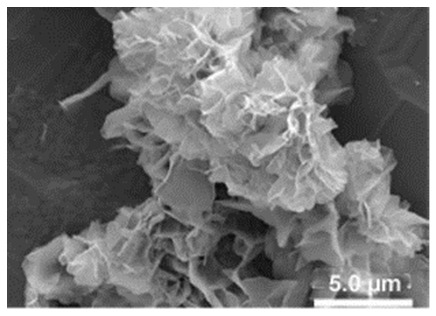	23. Temperature: Above T_t_, at 37 °C ELP/**Cu** hNFs 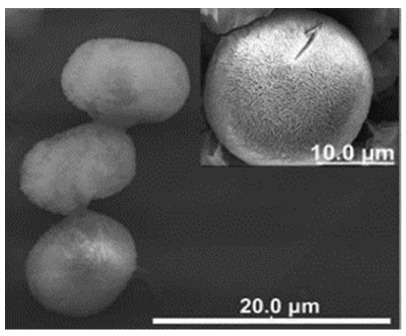	[93]
Different reaction temperature # Example 2	24. Temperature: 20 °C 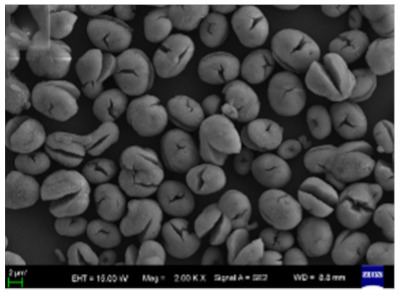	25. Temperature: 30 °C 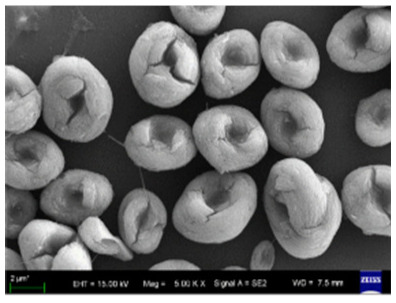	26. Temperature: 40 °C 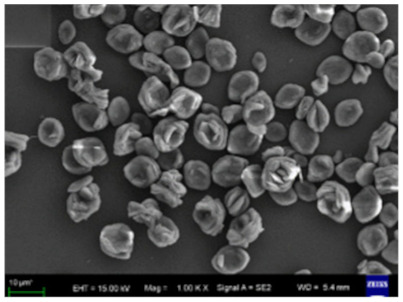	27. Temperature: 60 °C 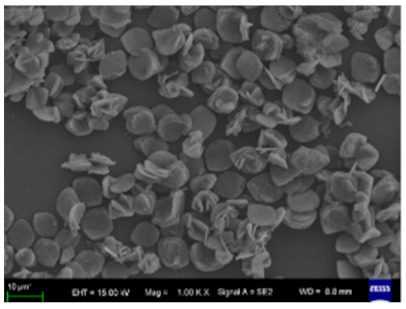	[35]
Different reaction temperature # Example 3	28. Temperature: 4 °C 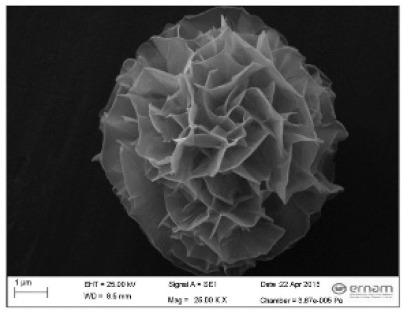	29. Temperature: 20 °C 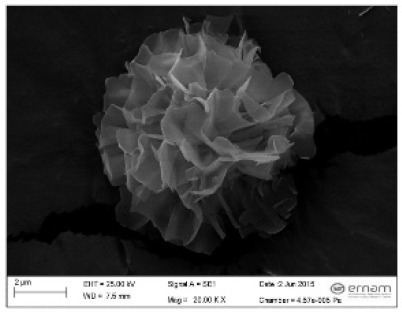	-	-	[51]
Reaction time	Morphology				
Different reaction time	30. Time: 2 h 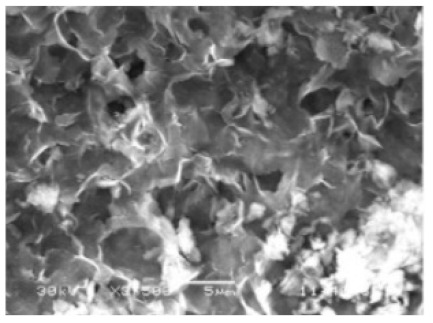	31. Time: 8 h 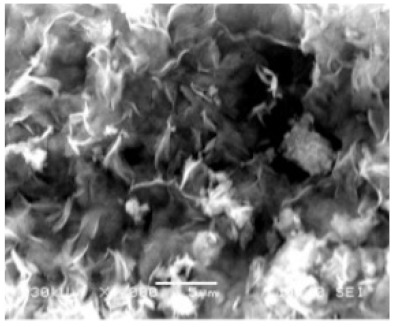	32. Time: 24 h 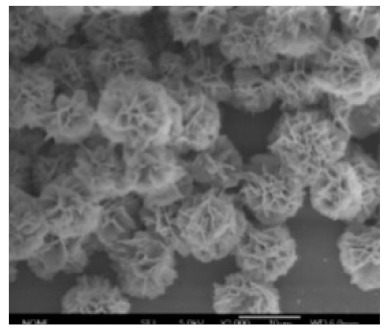	-	[39]

## Data Availability

Not applicable.
